# Isolation and Characterization of Ochrobactrum tritici for Penicillin V Potassium Degradation

**DOI:** 10.1128/mSphere.00058-20

**Published:** 2020-03-18

**Authors:** Peng Wang, Chen Shen, Kaili Xu, Qinqin Cong, Zhe Dong, Luwei Li, Jinfeng Guo, Jialin Lu, Shouxin Liu

**Affiliations:** a College of Chemical & Pharmaceutical Engineering, Hebei University of Science & Technology, Shijiazhuang, China; b State Key Laboratory Breeding Base–Hebei Province Key Laboratory of Molecular Chemistry for Drug, Hebei University of Science & Technology, Shijiazhuang, China; c Hebei Province Pharmaceutical Chemical Engineering Technology Research Center, Shijiazhuang, China; University of Minnesota

**Keywords:** antibiotic, bacterial residue, penicillin V potassium, biodegradation, *Ochrobactrum tritici*, biotransformation

## Abstract

Substantial concentrations of penicillin V potassium (PVK) have been found in livestock manure, soil, and wastewater effluents, which may pose potential threats to human health and contribute to the emergence of penicillin-resistant bacterial strains. In this study, bacterial strains capable of degrading PVK were isolated from sludge and characterized. Strain X-2 was selected for biodegradation of PVK. Based on morphological observations and 16S rRNA gene sequencing, strain X-2 was identified as an Ochrobactrum tritici strain. To enhance the PVK degradation ability of PVK, a whole-cell biodegradation process of Ochrobactrum tritici X-2 was established and optimized. In the whole-cell biodegradation process, the optimal temperature and pH were 30°C and 7.0, respectively. Under the optimized conditions, the degradation rate using 0.5 mg/ml PVK reached 100% within 3 h. During biodegradation, two major metabolites were detected: penicilloic acid and phenolic acid. The present study provides a novel method for the biodegradation of PVK using Ochrobactrum tritici strains, which represent promising candidates for the industrial biodegradation of PVK.

**IMPORTANCE** Substantial concentrations of penicillin V potassium (PVK) have been found in the environment, which may pose potential threats to human health and contribute to the emergence of penicillin-resistant bacterial strains. In this study, antibiotic-degrading bacterial strains for PVK were isolated from sludge and characterized. Ochrobactrum tritici was selected for the biodegradation of PVK with high efficiency. To enhance its PVK degradation ability, a whole-cell biodegradation process was established and optimized using Ochrobactrum tritici. The degradation rate with 0.5 mg/ml PVK reached 100% within 3 h. The potential biodegradation pathway was also investigated. To the best of our knowledge, the present study provides new insights into the biodegradation of PVK using an Ochrobactrum tritici strain, a promising candidate strain for the industrial biodegradation of β-lactam antibiotics.

## Abstract (precis)

Substantial concentrations of penicillin V potassium (PVK) have been found in the environment, which may pose potential threats to human health and contribute to the emergence of penicillin-resistant bacterial strains. In this study, antibiotic-degrading bacterial strains for PVK were isolated from sludge and characterized. Ochrobactrum tritici was selected for the biodegradation of PVK with high efficiency. To enhance its PVK degradation ability, a whole-cell biodegradation process was established and optimized using Ochrobactrum tritici. The degradation rate with 0.5 mg/ml PVK reached 100% within 3 h. The potential biodegradation pathway was also investigated. To the best of our knowledge, the present study provides new insights into the biodegradation of PVK using an Ochrobactrum tritici strain, a promising candidate strain for the industrial biodegradation of β-lactam antibiotics.

## INTRODUCTION

Antibiotics are some of the most widely used drugs for humans and other animals in clinical medicine and for growth promotion in animal production (1–3). The global consumption of antibiotics was reported to increase by 36% from 2000 to 2010 (4). However, the increased use of antibiotics has led to the accumulation of antibiotics in the environment in recent years (5). During the fermentation process for antibiotic production, some residual antibiotics are discharged into water (6). The accumulation of antibiotics in the environment has received much attention due to the impact of antibiotics on human health and the environment (7). Antibiotics in the environment can lead to the emergence of resistant pathogens and the spread of antibiotic resistance genes (8).

Penicillins are a commonly used groups of antibiotics that attack a wide range of bacteria; they include penicillin V potassium (PVK), a slow-onset antibiotic used to treat a number of bacterial infections (9). PVK is a broad-spectrum antibiotic that has been used to treat many types of mild to moderate bacterial infections, including scarlet fever, pneumonia, skin infections, and infections affecting the nose, mouth, or throat. Although it is less active against Gram-negative bacteria than other drugs, such as penicillin G, PVK is more acid stable than natural penicillins, allowing it to be taken orally without being decomposed by gastric acid. Furthermore, PVK is designed to have a good hydrolysis-resistant capability, strengthening its stability in treating bacterial infections (10). However, with the widespread use of PVK, substantial concentrations of PVK have been found in livestock manure, soil, and wastewater effluents, which may pose potential threats to human health and contribute to the emergence of penicillin-resistant bacterial strains. Therefore, it is imperative for researchers to identify ways to degrade PVK residues in the environment (11).

In general, it is very difficult to remove antibiotics by conventional physical or chemical treatments. Physical treatments usually use absorption or membrane techniques, which are often applied as complements to chemical or biological techniques. Common chemical treatments include the chlorination oxidation method and the Fenton oxidation method. Although these methods have the advantage of a high pollutant clearance rate, oxidants are prone to causing secondary environmental pollution. The low degradation rate of oxidants has attracted the attention of the scientific community, which has made considerable efforts to identify the right treatments to remove these emerging pollutants (12). In recent years, various methods to remove antibiotics from wastes have been explored, including chemical hydrolysis (13), membrane separation (14), activated carbon adsorption (15), and photodegradation (16). However, there are a number of drawbacks associated with these methods, including the formation of secondary toxic by-products and high operational costs, when conventional physical and chemical techniques are used. Therefore, it is of great significance to seek efficient and safe resource utilization strategies for the removal of antibiotic bacterial residues.

Microbiological degradation has become an effective way to address antibiotic contamination due to its high efficiency, low cost, simple operation, and other advantages (17). In recent years, various strains isolated from the environment have been chosen as candidates for the degradation of antibiotic residues. For example, microbial inocula obtained from rhizosphere sediments of plants derived from experimentally constructed wetlands have been designed for the treatment of livestock wastewaters contaminated with trace amounts of fluoroquinolones and cephalosporins (18). Furthermore, microbiotas previously acclimated to high levels of ciprofloxacin have been found to be able to utilize ciprofloxacin as their sole carbon and nitrogen source and to exhibit a much greater capacity for removal of ciprofloxacin than microbiotas not so acclimated (19). In addition, ammonia-oxidizing bacteria and nitrite-oxidizing bacteria have been used to remove micropollutants by nitrification processes attributed to the cometabolism of nitrifying sludge (20).

In this study, a highly efficient penicillin V potassium-degrading strain was isolated from activated sludge. Based on the analysis of fermentation conditions, the degradation conditions were optimized. The key intermediates in the degradation of PVK were identified by high-performance liquid chromatography (HPLC), mass spectrometry (MS), and ^1^H and ^13^C nuclear magnetic resonance (NMR) to explore their possible degradation pathways. An environmentally friendly treatment process for the degradation of bacterial PVK residue by whole-cell catalysis was explored and identified as a new method for antibiotic bacterial residue treatment.

## RESULTS

### Morphological features of the isolated strains.

Using PVK as the sole carbon source, four strains resistant to PVK were obtained by enrichment, acclimatization, and isolation. The strain numbers initially assigned were X-1, X-2, X-3, and X-4. Among these strains, only strain X-2 showed biodegradation activity for PVK (see Fig. S1 in the supplemental material). Therefore, X-2 was selected for further study.

10.1128/mSphere.00058-20.1 FIG S1 Biodegradation of PVK by selected bacterial strain X-2 detected in initial screening by HPLC. Download FIG S1, TIF file, 0.6 MB. Copyright © 2020 Wang et al. 2020 Wang et al. This content is distributed under the terms of the Creative Commons Attribution 4.0 International license.

**FIG 1:**
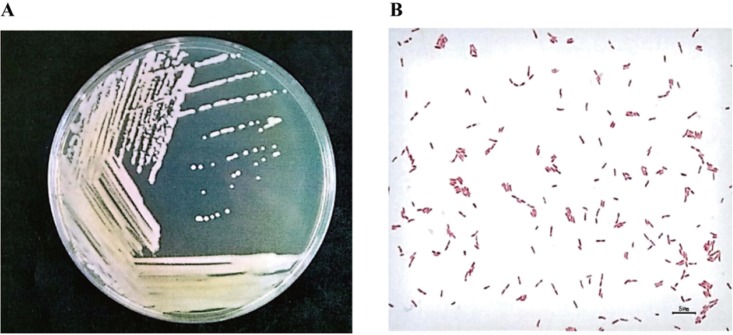
Morphological features of strain X-2. (A) Colony morphology of strain X-2; (B) morphological characteristics of strain X-2 under light microscopy.

X-2 cells were diluted, inoculated on solid medium, and cultured for 48 h at 31°C. The colonial morphology of X-2 (Fig. 1A) was as follows: light yellow, round, moist, opaque, and smooth. Figure 1B shows the morphology of X-2 cells observed under a microscope. The cells were rod shaped, 0.3 to 0.5 μm by 0.6 to 1.7 μm, and single or in pairs. The morphological features of two colonies suggested that X-2 was a Gram-negative bacterium.

### Physiological and biochemical characterization of strain X-2.

The physiological and biochemical reactions of strain X-2 were investigated. As shown in Table S2, positive results for l-pyrrolidinyl arylamine, l-valine, tyrosine arylamine, urease, d-tagatose, l-lactate alkalization, succinate alkali, glycine arylamine, and Ellman’s reagent were obtained.

### Gene sequencing and phylogenetic analysis of strain X-2.

**FIG 2:**
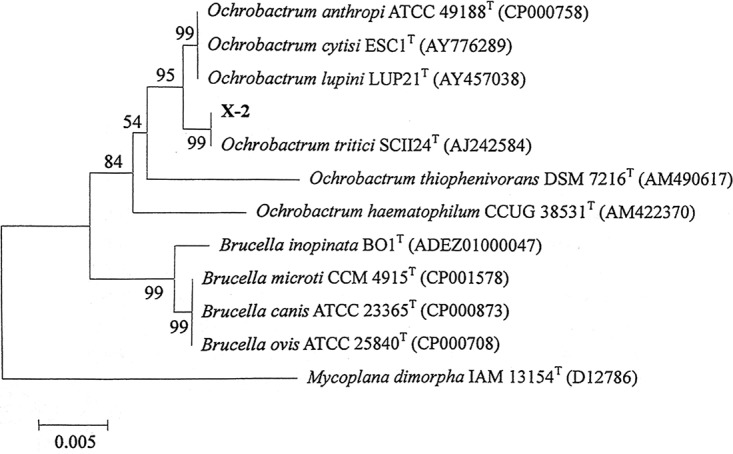
Phylogenetic analysis of strain X-2 in the neighbor-joining tree.

The 16S rRNA gene of isolate X-2 was amplified using the primers 27F and 1492R, resulting in a characteristic single band of approximately 1,490 bp (see Fig. S2). The 16S rRNA gene sequence of X-2 was aligned with the bacterial 16S rRNA gene database using BLAST modules. Strain X-2 was identified as belonging to the Ochrobactrum tritici subgroup on the basis of 16S rRNA gene sequencing and the phylogenetic tree. As shown in Fig. 2, the phylogenetic trees of the isolates, which were constructed with the neighbor-joining method using the Molecular Evolutionary Genetics Analysis (MEGA) 7.0 program, clearly demonstrated their evolutionary relationship with a group of *Ochrobactrum* species. The closest relative of X-2 was *O. tritici* strain SCII24 (AJ242584; 100% identity).

10.1128/mSphere.00058-20.2 FIG S2 Results of PCR amplification of bacterial isolate X-2. Download FIG S2, TIF file, 1.7 MB. Copyright © 2020 Wang et al. 2020 Wang et al. This content is distributed under the terms of the Creative Commons Attribution 4.0 International license.

### Optimal conditions for fermentation by whole-cell X-2.

**FIG 3:**
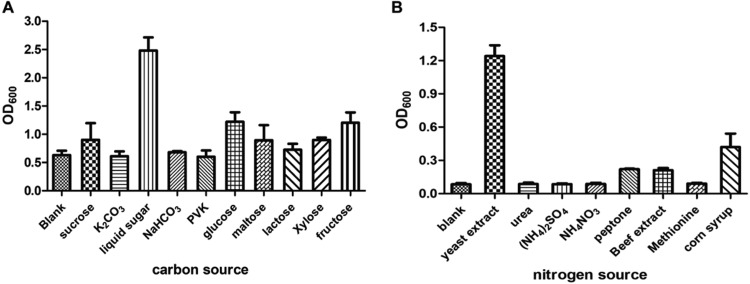
(A) Effect of different carbon sources on the growth of Ochrobactrum tritici X-2. The fermentation process was conducted at 30°C and pH 7.0 for 24 h. (B) Effect of nitrogen sources on the growth of strain Ochrobactrum tritici X-2. The reactions were conducted at 30°C and pH 7.0 for 24 h. Data are the means from three experiments, and error bars represent standard deviations.

Figure 3A shows that the bacterial strain *O. tritici* X-2 utilized organic carbon sources more efficiently than inorganic carbon sources, among which liquid sugar was chosen as the optimized carbon source. As shown in Fig. 3B, with liquid sugar as the carbon source, the cell density of strain *O. tritici* X-2 was high when yeast extract was used; therefore, yeast extract was selected as the nitrogen source for strain fermentation.

**FIG 4:**
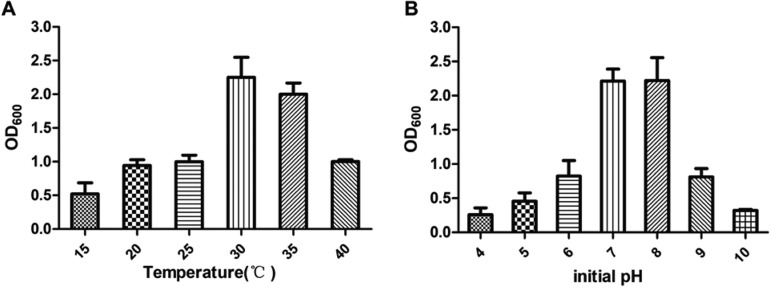
(A) Effect of temperature on the growth of Ochrobactrum tritici X-2. The fermentation process was conducted at pH 7.0 for 24 h by varying temperature from 15 to 40°C. (B) Effect of pH on the growth of strain Ochrobactrum tritici X-2. The reactions were conducted at 30°C for 24 h by using pH values from 4.0 to 10.0. Data are the means from three experiments, and error bars represent standard deviations.

As shown in Fig. 4A, when the temperature was 30°C to 35°C, *O. tritici* X-2 grew well. The initial pH value of the medium is also a key factor influencing the solubility of pollutants. As shown in Fig. 4B, when the pH was 6.8 to 7.2, the cell density of strain *O. tritici* X-2 reached the maximum value.

### Effect of temperature and initial pH on whole-cell biodegradation.

**FIG 5:**
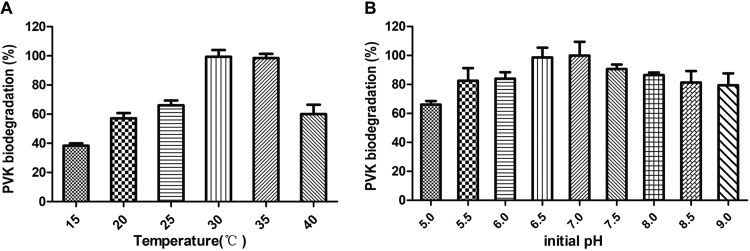
(A) Effect of temperature on the biodegradation of PVK by Ochrobactrum tritici X-2 whole-cell biotransformation. The reactions were conducted at pH 7.0 for 3 h by varying the temperature from 15 to 40°C. (B) Effect of pH on the biodegradation of PVK by Ochrobactrum tritici X-2 whole-cell biotransformation. The reactions were conducted at 30°C for 24 h by varying the pH value from 5.0 to 9.0. Data the means from three experiments, and error bars represent the standard deviations.

The effects of different temperature ranges on the biodegradation of PVK by Ochrobactrum tritici X-2 is shown in Fig. 5A. The optimum temperature for biodegradation of PVK by resting cells of Ochrobactrum tritici X-2 was 30 to 35°C. When the reaction temperature was higher than 35°C or lower than 25°C, the biodegradation rate began to decrease significantly.

The effect of initial pH on the biodegradation of PVK was evaluated for 3 h, and the results are shown in Fig. 5B. The optimal pH for PVK biodegradation by Ochrobactrum tritici X-2 was 6.5 to 7.5.

### Identification of biodegradation metabolites.

To analyze the potential catalytic pathway of Ochrobactrum tritici X-2 for the biodegradation of PVK, whole-cell assays were performed with PVK as the substrate. Product formation was detected by HPLC analysis with a diode array detector. Two major metabolites were detected in the HPLC analysis. Both metabolites were derived from cultures cultured with PVK for 48 h, and no peaks corresponding to these metabolites were observed for the negative control (no microorganisms).

**FIG 6:**
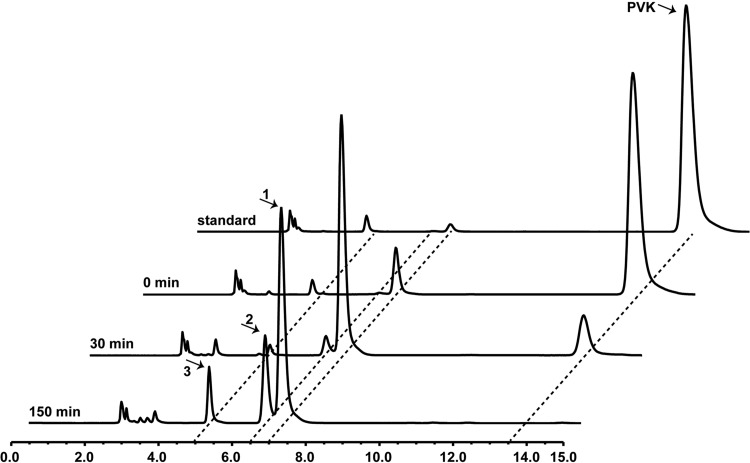
HPLC analysis of biodegradation of PVK by Ochrobactrum tritici X-2.

**FIG 7:**
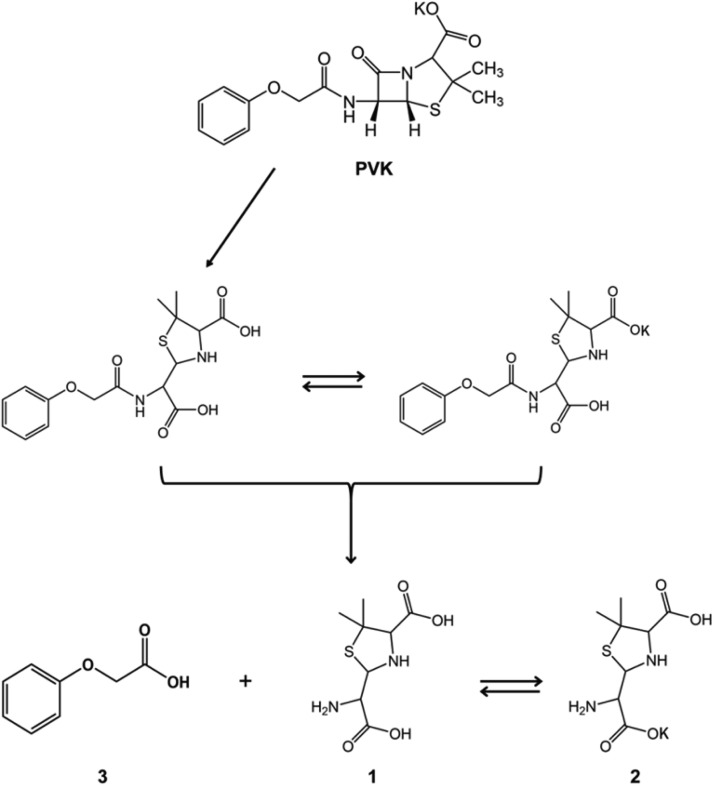
Biodegradation profiles of PVK by Ochrobactrum tritici X-2.

As shown in Fig. 6 and Table 1, HPLC analysis of PVK biodegradation by Ochrobactrum tritici X-2 revealed three major peaks (retention times of 6.857 min, 6.422 min, and 4.902 min) in addition to the substrate peak (13.280 min). The new peak was not detected following the reaction with the negative control. To determine the structures of the biodegradation products during the course of the biodegradation, the products were isolated from the crude extracts of the reaction mixtures and were further analyzed by MS and NMR. MS analyses revealed the accumulation of products 1 and 2 (retention times of 6.857 min and 6.422 min) with *m/z* values of 217.25 [M + H]^+^ and 255.11 [M + H]^+^, respectively. Based on the NMR analysis, these two degradation products were speculated to be penicilloic acid and penicilloic acid potassium, as shown in Fig. 7. MS analyses revealed the accumulation of product 3 (retention time of 4.902 min) with an *m/z* of 153.03 [M + H]^+^. The NMR spectra of this compound were the same as those of authentic phenoxyacetic acid. The deduced biodegradation pathway of PVK by *O. tritici* X-2 is shown in Fig. 7.

**TABLE 1:** HPLC and MS analysis of PVK metabolism biocatalytic profile

Compound	HPLC retention time (min)	*m*/*z* ([M + H]^+^)
PVK	13.268	389.49
Metabolism product 1	6.875	217.25
Metabolism product 2	6.422	255.11
Metabolism product 3	4.889	153.03

## DISCUSSION

In recent years, substantial concentrations of PVK have been found in the environment, which may contribute to the emergence of penicillin-resistant bacterial strains and pose potential threats to human health, such as infection with antibiotic-resistant strains. Therefore, it is imperative for researchers to identify ways to degrade PVK residues in the environment. Several methods have been used for PVK degradation. For example, penicillin V can be degraded under visible light when TiO_2_ is used as a photocatalyst (21). It has also been reported that simulated sunlight is able to degrade PVK (22). In addition, biodegradation methods have also been used for the treatment of PVK. Purified enzymes are one of the most promising candidates for β-lactam antibiotic degradation. For example, β-lactamase immobilized on Fe_3_O_4_ magnetic nanoparticles has been used for the degradation of β-lactam antibiotics in wastewater (23). In addition, whole bacterial cells can be used for β-lactam antibiotic degradation. The β-lactam-degrading bacteria most commonly reported in the literature are mainly *Bacillus* species, such as Bacillus cereus (11, 24). To the best of our knowledge, this study is the first report of whole-cell biodegradation of PVK by an *Ochrobactrum* species. In this study, an Ochrobactrum tritici isolate was identified as a PVK-degrading strain with high efficiency. Members of this genus are saprophytic soil and water bacteria, and many isolates of this genus are responsible for the biotransformation or biodegradation of a wide variety of toxic organic pollutants. In previous studies, Ochrobactrum tritici was used for the biodegradation of dichlorvos (25), nicotine (26), lindane (27), naphthalene (28), and kraft lignin (29). It has also been reported that Ochrobactrum tritici can degrade phenol (30) and a synthetic pyrethroid (31).

Two products were detected by HPLC in the biodegraded extracts. One product was identified as phenoxyacetic acid. The deduced whole process of biodegradation of PVK by Ochrobactrum tritici X-2 is shown in Fig. 7. The β-lactam of PVK was first hydrolyzed to form the β-lactam ring-cleaved product phenoxyethyl penicilloic acid; this step was most likely catalyzed by β-lactamase. Then, this product was further hydrolyzed to form penicilloic acid or penicilloic acid potassium and phenoxyacetic acid. This step was probably catalyzed by penicillin V acylase (32).

To predict the potential candidate enzymes driving the biodegradation of PVK, the NCBI database was searched, and several proposed β-lactamase-like proteins in Ochrobactrum tritici species were found. The GenBank numbers of these β-lactamase like proteins from Ochrobactrum tritici are SME85995.1, SME85999.1, and SME86000.1. Furthermore, the potential enzymes in Ochrobactrum tritici species that cleave the amino bond were also searched in the NCBI database. Three enzymes designated amide C-N hydrolases were identified as potential enzymes cleaving the amino bond of penicillin V. The accession numbers of these three enzymes in the NCBI database are WP_151653093.1, WP_151558213.1, and WP_151643919.1. In our future studies, the whole genome of *Ochrobactrum* X-2 will be sequenced. The candidate enzymes in Ochrobactrum tritici X-2 for PVK biodegradation elucidated here may aid the development of an enzymatic degradation system.

Regarding PVK degradation technology, degradation-separation coupling technology has been considered, and the combination of *in situ* product removal (ISPR) technology and cell and enzyme immobilization technology has been discussed (33, 34). In future work, we will study the structure-activity transformation and transport of key degrading enzymes under a new ISPR model and aim to elucidate the law of trans-scale matter and energy transfer in this biocatalytic system. Such work will provide a theoretical basis for the effectiveness of the treatment processes for wastewater containing β-lactam antibiotics.

In conclusion, in the present study, four strains with the ability to degrade PVK were screened from activated sludge samples by the classical bacterial screening method. Among them, strain X-2 was found to be capable of growth with PVK as the sole carbon source and exhibited penicillin-degrading activity. According to its colony morphological structure, physiological and biochemical characteristics, and 16S rRNA gene sequence analysis, strain X-2 was identified as Ochrobactrum tritici. When liquid sugar was used as the carbon source, yeast extract was used as the nitrogen source, and the initial pH was 6.8 to 7.2, the cell density of Ochrobactrum tritici X*-2* reached its maximum. The major biodegradation metabolites were analyzed, and two major metabolites were identified. An important future research direction is the identification of microbial remediation methods to reduce antibiotic pollution in the environment.

## MATERIALS AND METHODS

### Reagents and culture medium.

Penicillin V potassium (PVK) was provided by North China Pharmaceutical Group Corporation, China. All of the other chemicals and reagents used in this study were of analytical grade quality and are available commercially.

Mineral salts basal medium (MSM) containing 1.60 g/liter K_2_HPO_4_, 0.40 g/liter KH_2_PO_4_, 0.20 g/liter MgSO_4_·7H_2_O, 0.03 g/liter CaCl_2_·2H_2_O, 0.02 g/liter FeCl_3_·6H_2_O, 0.50 g/liter NH_4_NO_3_, and 0.50 g/liter yeast extract was used for enrichment culture. The pH of the medium was adjusted to 7.0 with H_3_PO_4_, and the mixture was autoclaved at 121°C for 1 h. To produce solid medium, 2.0% agar (wt/vol) was added.

### Enrichment of bacterial strains.

Sludge samples were collected from a pharmaceutical factory in Shijiazhuang, China. The collected sludge was placed in sealed sterile bags and maintained at a temperature of −20°C before the experiment.

Enrichment of the bacterial strains was carried out through a series of dilutions and incubations. First, 1 g of collected soil was suspended in 9 ml of sterile distilled water and agitated for 1 min. This homogenized soil extract solution was then diluted by adding 1 ml of the solution to 9 ml of sterile purified water, and the process was repeated six times to achieve a 10^6^-fold dilution. One milliliter of the diluted solution was then added to 49 ml of sterile LB medium containing 20 μg/ml PVK as the sole carbon source, and the mixture was incubated at 30°C and shaken at 150 rpm for 6 or 7 days until an optimal optical density was reached.

### Isolation and qualitative screening of PVK-degrading bacteria.

Isolation and purification of the strain were performed after enrichment. Ten grams was accurately weighed into 100 ml of basic inorganic salt medium, and 0.2 g/liter PVK was added as an inducer. The culture was shaken at 37°C and 150 rpm for 24 h. Then, 10 ml of the above-described fermentation solution was transferred to 100 ml of fresh medium and incubated under the same conditions for 2 days. The obtained fermentation broth was diluted to a series of concentrations from 10^−1^ to 10^−6^ by a 10-fold gradient dilution method; 0.1 ml of each bacterial solution was used to uniformly coat solid medium containing 20 mg/ml PVK and incubated at 37°C, which was kept constant for 2 days. A single colony was selected and inoculated on solid medium for rescreening and purification.

To qualitatively determine if an isolated bacterial strain was capable of degrading PVK, a whole-cell biodegradation experiment was conducted. Cultures of the selected strains were grown in 10 ml of mineral salt basal medium in 50-ml Erlenmeyer flasks with shaking at 150 rpm at 30°C for 48 h. Then, the fermentation broth was centrifuged at a speed of 5,000 rpm for 10 min. The bacterial pellet was resuspended in tap water and was adjusted to 15 g/liter (wet weight). Next, 0.2 g/liter PVK was added, and the suspension was shaken at 30°C and 160 rpm for 24 h. HPLC was used to determine whether the selected strain could degrade PVK.

A piece of filter paper containing 10 μg of PVK was applied to the surface of agar that had been inoculated with a bacterial strain. As the diffusion distance of PVK in the agar increased, the PVK concentration decreased logarithmically to a certain concentration below which the bacterium did not grow; thus, a transparent antimicrobial circle was formed on the filter paper. The size of this inhibition zone reflected the sensitivity of the test bacterium to PVK: the smaller the circle, the more effective the bacterium in degrading PVK.

### Morphological observation of isolated strains.

Gram staining was used to observe the morphology of the isolated bacterial strains as a means of preliminary identification, following the method reported in reference 20. The observed morphological features included color, opaqueness, and surface texture of the bacterial colony, as these features can potentially aid the visual identification of bacterial strains.

The physiological and biochemical characteristics were mainly analyzed using Bergey’s bacterial identification manual as a reference (35).

### Gene sequencing and phylogenetic analysis.

Strain X-2 was streaked on a solid medium, and then the plate was placed in a 37°C incubator for inversion culture. The culture was used for the amplification of the bacterial 16S rRNA gene by PCR. Two universal 16S rRNA gene primers (F27 [AGTTTGATCMTGGCTCAG] and R1492 [GGTTACCTTGTTACGACTT]) were employed. Culture samples of 25 μl were prepared. Each sample was composed of 0.5 μl of bacterial culture as the template DNA, 7.5 μl of 2× *Taq* PCR master mix (containing 0.2 U *Taq* DNA polymerase/μl, 250 μM deoxyribonucleoside triphosphate, and 2.5 μl of 10× buffer with Mg^2+^ [TaKaRa, Tianjin, China]), 0.5 μl of primer (10 μM), and 19.8 μl of double-distilled H_2_O. The PCR conditions were as follows: primary denaturation at 94°C for 4 min; 30 cycles of denaturation at 94°C for 45 s, annealing at 55°C for 45 s, and extension at 72°C for 60 s; and an additional reaction for 10 min at 72°C. The PCR products were detected on 1.5% agarose gel to confirm their purity and size. The PCR products were sent to Sangon Biotech (Shanghai, China) for sequencing.

The 16S rRNA gene sequences were compared with other 16S rRNA gene sequences available in GenBank by using the Basic Local Alignment Search Tool (BLASTN) program and BLASTn database and aligned with similar sequences by using multiple sequence alignment software (21). A phylogenetic tree was constructed by applying the neighbor-joining method using the MEGA 7.0 program based on Kimura-2 parameters with 1,000 replicates of bootstrap values.

### Whole-cell biodegradation of PVK.

Cultures of the PVK-degrading strains were grown in 100 ml of mineral salt basal medium in 250-ml Erlenmeyer flasks. The mineral salt basal medium is described in “Reagents and culture medium” above and was autoclaved at 121°C for 15 min before use. Cultures were incubated with shaking at 150 rpm at 30°C for 48 h.

The fermentation broth was centrifuged at a speed of 5,000 rpm with a high-speed centrifuge for 10 min. The supernatant was removed, and the bacterial pellet was washed with sterile saline. Subsequently, the collected precipitate was adjusted to 15 g/liter (wet weight), inoculated into 100 ml of tap water containing 0.2 g/liter PVK, and shaken at 30°C and 160 rpm. HPLC was used to detect the content of PVK in the sample every 1 h to evaluate its degradation. Each experiment was repeated three times and a control group was established in each.

The PVK biodegradation percentage was calculated as follows: [(PVK_0_ − PVK*_t_*)/PVK_0_] × 100, where PVK_0_ and PVK*_t_* are the PVK concentrations (in milligrams per liter) in the feed solution and in the mixed liquor-supernatant sample taken at time *t* of the biotreatment, respectively.

### Optimization of conditions for strain fermentation and whole-cell biodegradation.

The carbon source, nitrogen source, temperature, and initial pH were optimized for the fermentation of PVK-degrading strains. Various carbon sources, such as glucose, fructose, maltose, lactose, xylose, sucrose, liquid sugar, PVK, sodium bicarbonate, and potassium carbonate, and various nitrogen sources, such as urea, ammonium sulfate, ammonium nitrate, yeast extract, peptone, beef extract, methionine, and corn syrup, were used for screening. The concentration of the liquid sugar was 10 ml/liter, and the concentration of the other carbon sources was 1 g/liter. The concentration of the screened nitrogen sources was 0.5 g/liter. The effect of temperature on fermentation by the PVK-degrading strain was determined over a temperature interval ranging from 15 to 40°C. The effect of initial pH on fermentation by the PVK-degrading strain was determined over a pH range of 4.0 to 10.0.

Next, the effect of temperature on whole-cell degradation was determined over a temperature interval ranging from 15 to 40°C. The effect of the initial pH on whole-cell degradation was determined over a pH range of 4.0 to 9.0.

### Characterization of degradation intermediates by HPLC and NMR analysis.

Analysis of the degradation products was performed using an Agilent 1260 high-performance liquid chromatography (HPLC) instrument. The degradation products and substrate PVK were separated on a reversed-phase C_18_ column (4.6 by 250 mm^2^, 5 μm; Ultimate XB-C18) at a flow rate of 1 ml/min. Mobile phase A was 0.5 M potassium dihydrogen phosphate (with pH adjusted to 3.5 using phosphate), mobile phase B was deionized water, and mobile phase C was methanol. The ratio of mobile phases A, B, and C was set as 1:4:5.

For NMR analysis of the purified products, compounds were dissolved in a D_2_O solution and analyzed using a Bruker AV-300 spectrometer for one-dimensional ^1^H NMR and ^13^C NMR. For the detailed NMR data of the identified products, see Table S1.

10.1128/mSphere.00058-20.3 TABLE S1 NMR data for metabolism products 2 and 3. Download Table S1, DOCX file, 0.01 MB. Copyright © 2020 Wang et al. 2020 Wang et al. This content is distributed under the terms of the Creative Commons Attribution 4.0 International license.

10.1128/mSphere.00058-20.4 TABLE S2 Physiological and biochemical reactions of bacterial isolate X-2. Download Table S2, DOCX file, 0.02 MB. Copyright © 2020 Wang et al. 2020 Wang et al. This content is distributed under the terms of the Creative Commons Attribution 4.0 International license.
