# Supplementary figures and images for: Isolation and Characterization of Ochrobactrum tritici for Penicillin V Potassium Degradation
Source: mSphere. 2020 Mar 18;5(2):e00058-20. doi: 10.1128/mSphere.00058-20 (PMC7082136; doi:10.1128/mSphere.00058-20)

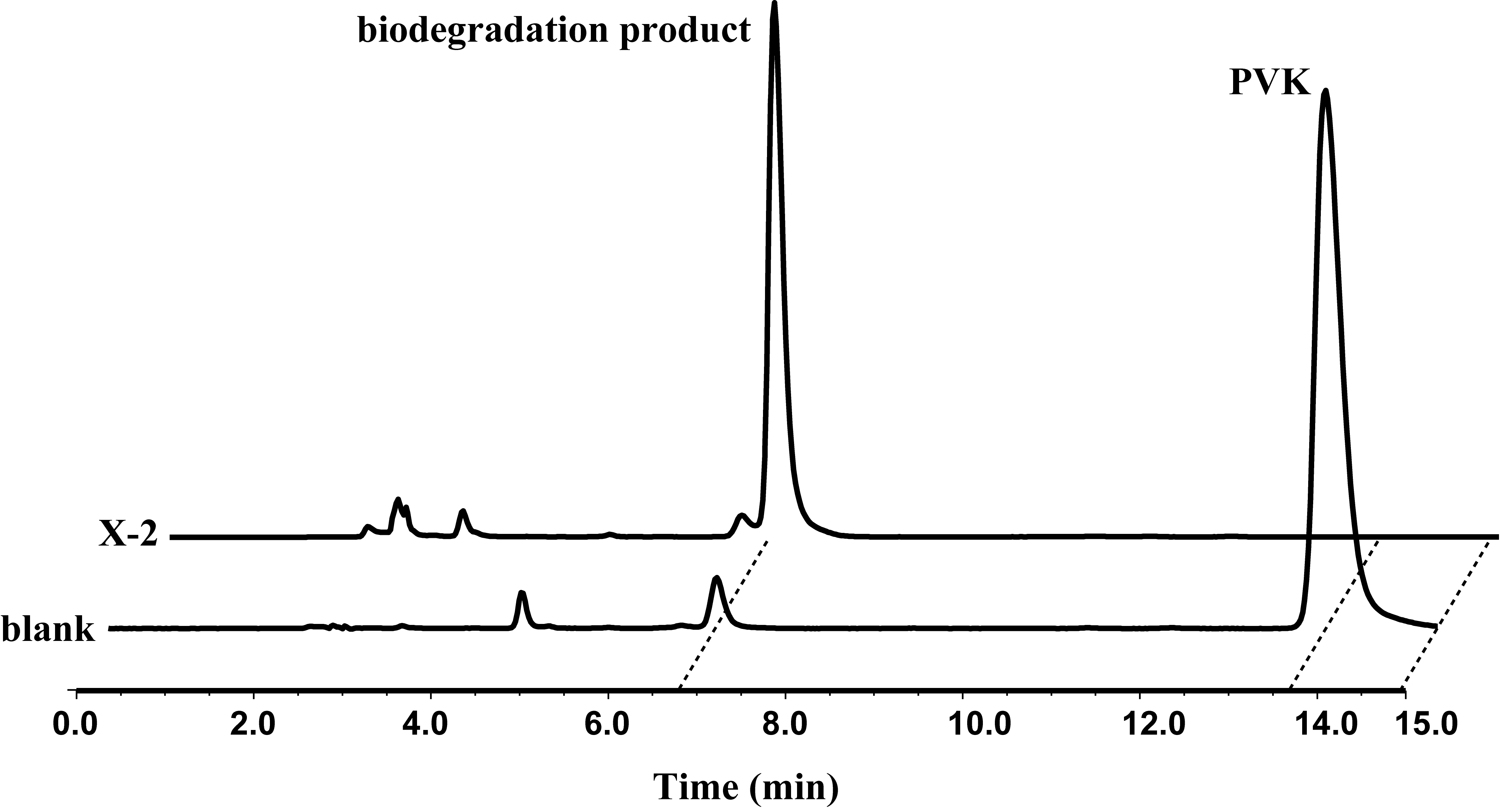

Supplement: FIG S1 [file mSphere.00058-20-sf001.tif]

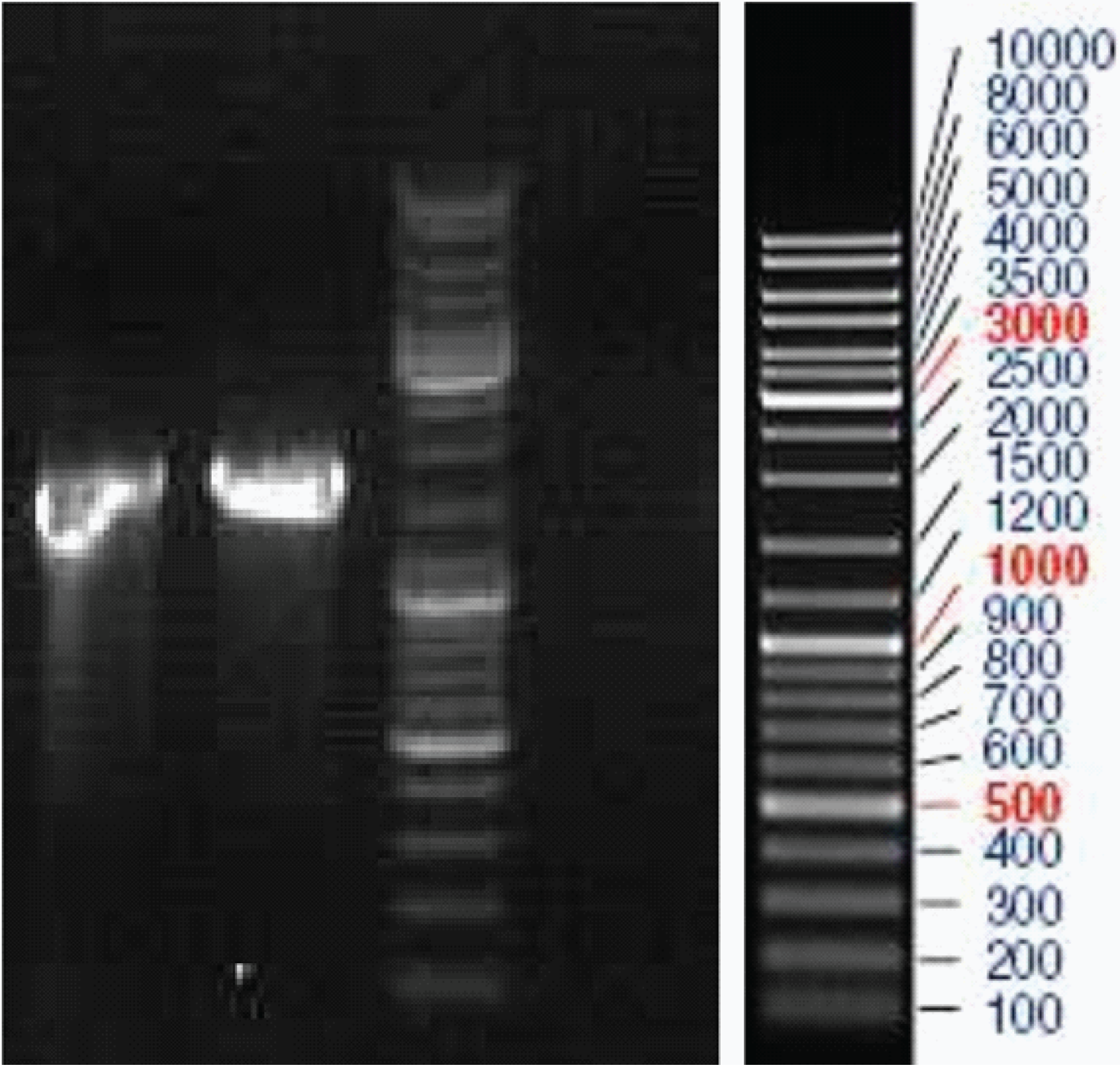

Supplement: FIG S2 [file mSphere.00058-20-sf002.tif]
