# Supplementary material for: Isolation and Characterization of Ochrobactrum tritici for Penicillin V Potassium Degradation
Source: mSphere. 2020 Mar 18;5(2):e00058-20. doi: 10.1128/mSphere.00058-20 (PMC7082136; doi:10.1128/mSphere.00058-20)
Supplement: TABLE S1 [file mSphere.00058-20-st001.docx]

**Table S1 NMR data of the products of 2 and 3**

| **product** | ^1^HNMR(500MHz,D_2_O) | ^13^CNMR(125MHz,D_2_O) |
| --- | --- | --- |
| **2** | δ:7.39-7.36(m,2H),  7.071-7.042(t,J=14.51Hz,1H),  6.892-6.966(d,J=8.0Hz,2H),  4.50(s,2H). | δ:171.70, 158.42, 129.46(2C), 121.44, 114.61(2C), 64.79. |
| **3** | δ: 5.06–5.05 (d, J= 4.5 Hz, 1H);  4.85–4.83 (d, J=10.0 Hz, 1H);  3.97–3.96 (d, J=4.0 Hz, 1H);  3.64–3.62 (d, J=10.0 Hz, 1H);  3.56 (s, 2H);  3.47 (s, 1H);  3.19 (s,1H);  1.45 (s, 3H);  1.44 (s, 3H);  1.19 (s, 3H);  1.13(s, 3H). | δ: 176.34, 174.38, 171.85, 171.20, 74.47, 74.00, 63.59, 63.29, 59.31, 58.68, 56.80, 56.39, 29.29, 26.06, 26.02, 25.94. |
