# Supplementary material for: Isolation and Characterization of Ochrobactrum tritici for Penicillin V Potassium Degradation
Source: mSphere. 2020 Mar 18;5(2):e00058-20. doi: 10.1128/mSphere.00058-20 (PMC7082136; doi:10.1128/mSphere.00058-20)
Supplement: TABLE S2 [file mSphere.00058-20-st002.docx]

**Table S2 physiological and biochemical reactions of X-2**

| **Item** | ***+ /-*** | **Item** | ***+/-*** |
| --- | --- | --- | --- |
| Alanine - phenylalanine - proline aromaminase | ***-*** | Adonitol | ***+*** |
| D-cellobiose | ***-*** | β-galactosidase | ***-*** |
| Glutamine aromatase pNA | ***-*** | D-glucose | ***-*** |
| β-glucosidase | ***-*** | D-maltose | ***-*** |
| β-xylosidase | ***-*** | β-alanine arylamine pNA | ***-*** |
| Ancient sugar | ***-*** | Tyrosine arylamine | ***+*** |
| sucrose | ***-*** | D-tagatose | ***+*** |
| Malonate | ***-*** | 5-keto-gluconate | ***-*** |
| Succinate alkali | ***+*** | β-N-acetylgalactosidase | ***-*** |
| Glycine arylamine | ***+*** | Ornithine decarboxylase | ***-*** |
| Coumaric acid | ***-*** | β- glucuronidase | ***-*** |
| L-malate assimilation | ***-*** | ELLMAN reagent | ***+*** |
| L-pyrrolidinyl arylamine | ***+*** | L-arabitol | ***-*** |
| H2S production | ***-*** | β-N-acetylglucosaminidase | ***-*** |
| γ-glutamyltransferase | ***-*** | Glucose fermentation | ***-*** |
| D-mannitol | ***-*** | D-mannose | ***-*** |
| L-valine arylamine | ***+*** | Lipase | ***-*** |
| Urease | ***+*** | D-sorbitol | ***-*** |
| D-trehalose | ***-*** | Citrate (sodium) | ***-*** |
| L-lactate alkalization | ***+*** | Alpha-glucosidase | ***-*** |
| Alpha-galactosidase | ***-*** | Phosphatase | ***-*** |
| Lysine decarboxylase | ***-*** | O/129 resistance | ***-*** |
| L-histidine assimilation | ***-*** | L-lactate assimilation | ***-*** |
| Glutamate-glycine-arginine arylamine | ***-*** |  |  |

“+”，positive；“-”，negative
